# Buried in Sands: Environmental Analysis at the Archaeological Site of Xiaohe Cemetery, Xinjiang, China

**DOI:** 10.1371/journal.pone.0068957

**Published:** 2013-07-22

**Authors:** Jin-Feng Li, Idelisi Abuduresule, Francis M. Hueber, Wen-Ying Li, Xin-Jun Hu, Yue-Zhuo Li, Cheng-Sen Li

**Affiliations:** 1 State Key Laboratory of Systematic and Evolutionary Botany, Insititue of Botany, Chinese Academy of Sciences, Beijing, China; 2 Xinjiang Cultural Relics and Archaeology Institute, Urumchi, China; 3 Department of Paleobiology, Smithsonian Institution, Washington, District of Columbia, United States of America; 4 Sanya Museum of Natural History, Sanya, China; University of Oxford, United Kingdom

## Abstract

Palynomorphs extracted from the mud coffins and plant remains preserved at the archaeological site of Xiaohe Cemetery (Cal. 3980 to 3540 years BP) in Lop Nur Desert of Xinjiang, China were investigated for the reconstruction of the ancient environments at the site. The results demonstrate that the Xiaohe People lived at a well-developed oasis, which was surrounded by extensive desert. The vegetation in the oasis consisted of *Populus* *,* *Phragmites* *, Typha* and probably of Gramineae, while the desert surrounding the oasis had some common drought-resistant plants dominated by *Ephedra* , *Tamarix* , *Artemisia* and Chenopodiaceae. This present work provides the first data of the environmental background at this site for further archaeological investigation.

## Ethics Statement

All necessary permits were obtained for the described field studies and were granted by the Xinjiang Cultural Relics and Archaeology Institute.

## Introduction

In the past Xinjiang formed an important bridge connecting the Eastern and Western races of Eurasian continents and became famous for the ancient Silk Road going to Central Asia and Eastern Europe from China.

Consequently, the archaeological discoveries in this area are always of great interest (e.g. Loulan City). Signs of human activities can be traced in Xinjiang for 10,000 years [1]. Stone tools discovered at the site of Astana are nearly 5000 years old [2]. Many cemeteries were discovered since the 1970s, such as the Gumugou Cemetery (around 3800 B.P.) [3], Wufu Cemetery in the Hami District with an age of nearly 3300-3000 years old [4]. During the period from 3000 to 2000 B.P., a group of people lived in the Turpan Basin and adjacent area, and their different cemeteries, such as the Yanghai Tombs (ca. 2800 B.P.) [5], Yuergou Site (2400-2300 B.P.) [1], as well as many much younger sites, reveal much about the lives and beliefs of these peoples.

Many mummies were found well-preserved in this area, owing to the dryness of the desert and the desiccation of the corpses [6]. The fantastic mummies and the delicate relics from the archaeological sites in Xinjiang, including the artifacts and crops, can tell us amazing stories: what the world looked like at any given point in time and space [7]. The plant remains found at these sites provided an opportunity to study the ancient plants and their utilization by local people, as well as their bearing on environmental changes in the past. Some archaeobotanical researches have been done in Xinjiang in the past few years, mainly focusing on the relationship between plants and people [5,8–10] and also on the environmental data extracted from artifacts [11,12]. In the present contribution, the palynomorphs extracted from the mud coffins and plant remains found at Xiaohe Cemetery are investigated comprehensively for the reconstruction of the historical environments in Xiaohe.

### Site description

The Xiaohe (“Small River”) Cemetery was first discovered in 1911 by an aboriginal hunter named Ördek who played a part in Dr. Sven Hedin’s discovery of the Loulan ruins around 1910-1911 [13]. Two decades later, a Swedish archaeologist Folke Bergman, coined the name for this graveyard, and excavated 12 burials guided by Ördek in 1934 [13]. After that, the cemetery was forgotten for more than sixty years until the Relics and Archaeology Institute of Xinjiang Uygur Autonomous Region excavated this graveyard in detail in 2002 [14]. The rediscovery of Xiaohe Cemetery was considered to be one of the top ten archaeological discoveries in 2004 in China. About 170 tombs have been excavated since then, but unfortunately many of them were destroyed by treasure hunters.

**Figure 1:**
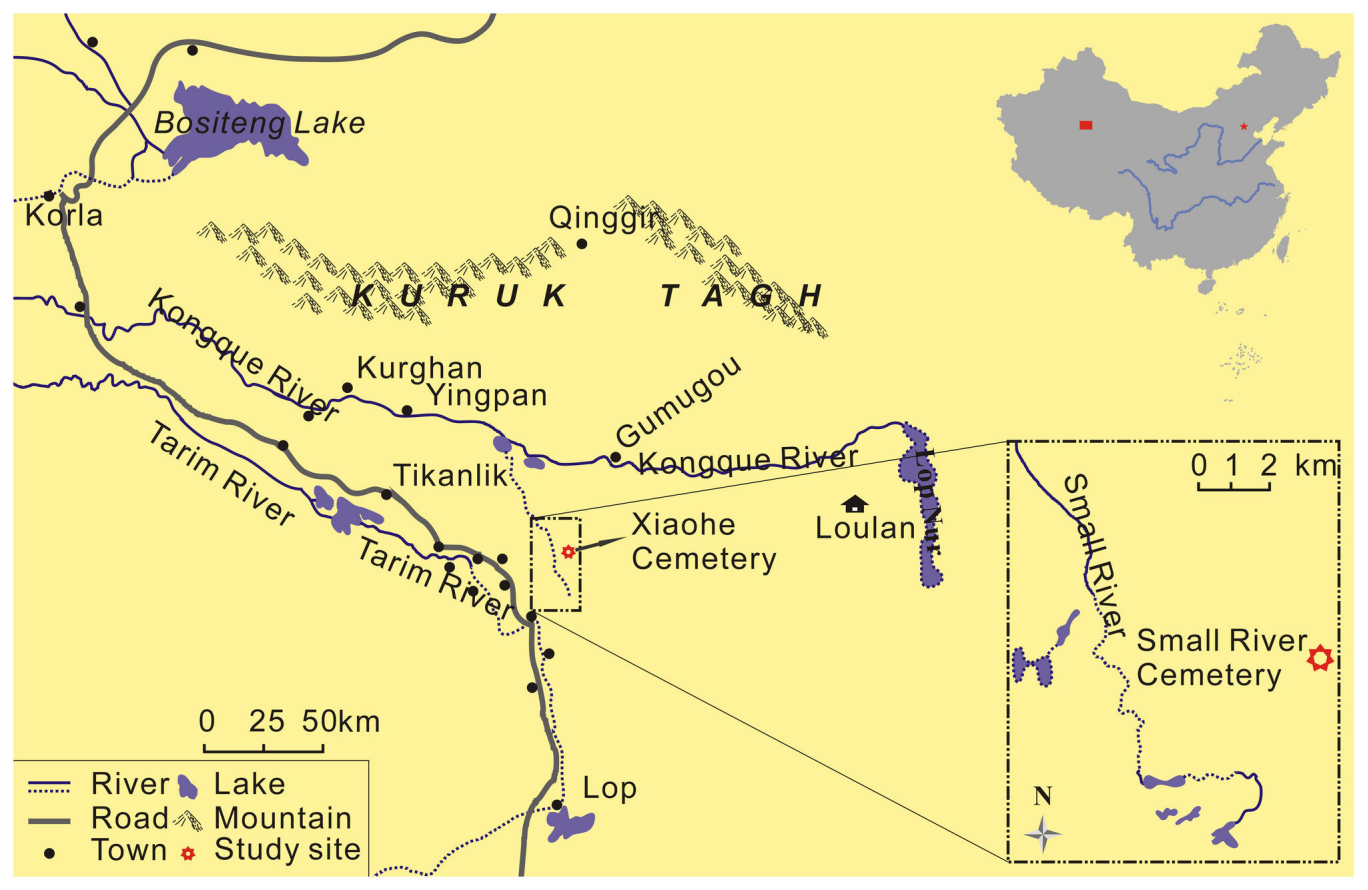
Map showing the Lop Region and the location of Xiaohe Cemetery (modified from [13]).

**Figure 2:**
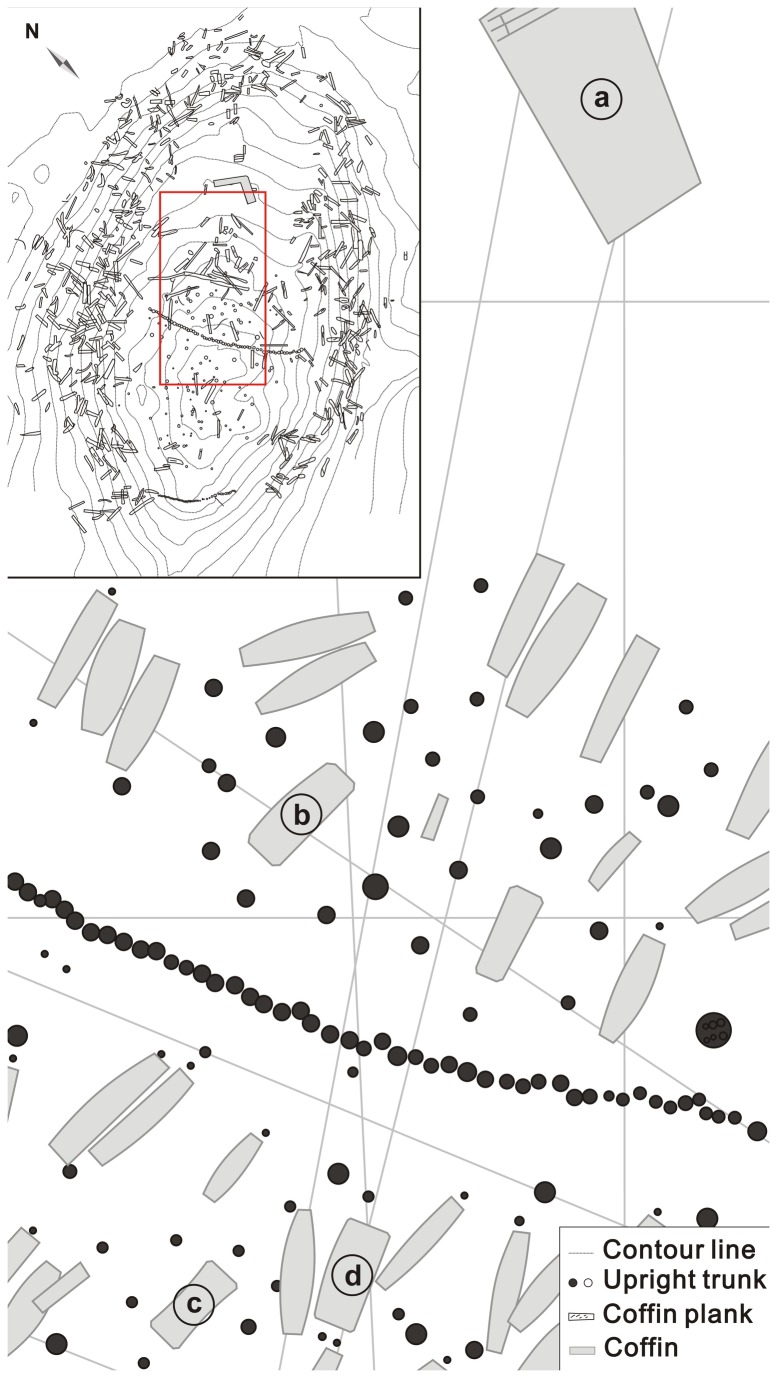
Plan diagram of the Xiaohe Cemetery. (a) mud coffin BM28; (b) mud coffin BM 1; (c) mud coffin M100; (d) mud coffin M75 (plan in top left corner revised from [14]).

The Xiaohe Cemetery, with an area of about 2500 m^2^, is some 4 km away from the Small River (Xiaohe, in Chinese), a downstream branch of Kongque River in Lop Desert (Figure 1) [15], and also about 175 km east of the Loulan ancient city in Xinjiang [14]. With its hillock shape the Xiaohe Cemetery forms a well-defined landmark on the flat desert. The top of the cemetery possesses many upright wild poplar poles and more fallen ones [13] (Figure 2). These poles were thought to be the remains of a house which had lost its roof a long time ago [13]. Two main kinds of trunks stood in the cemetery, i.e. the multi-prismatic shaped poles (= pole monuments in Bergman, 1939 [13]) are all placed in front of females’ tombs and the oar-shaped ones (= oar-like monuments in Bergman, 1939 [13]) in front of those of males. Some archaeologists inferred that these poles were the symbols of fertility worship of Xiaohe People [13]. The multi-prismatic shaped poles represent the phallus and the oar-shaped ones represent a vulva.

**Figure 3:**
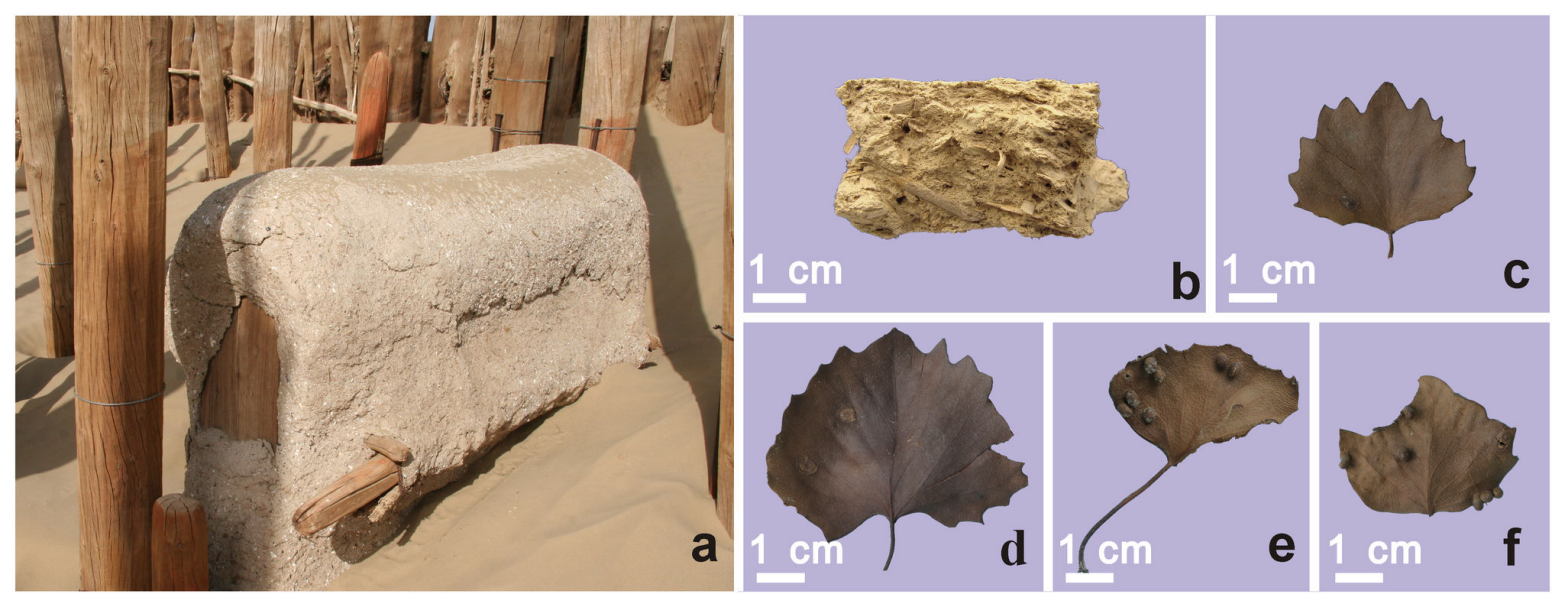
The mud coffin M75 (a); sample collected from the coffin (b); and the leaves of *Populus* *euphratica* found in the grave (c to f).

Most of the coffins, which are made from the wood of *Populus* *euphratica* Oliv., have an elliptical shape. However, some coffins are rectangular in shape and covered by a layer of mud. These are called “mud coffins” (Figure 3a). The result of ^14^C dating revealed that the age of the lowest layer of Xiaohe Cemetery is 3980 ± 40 yr BP [16], which is the oldest archaeological dating record in Xinjiang. Desiccated wheat grains from the cemetery were dated to approximately 3760–3540 yr BP [17]. Hence, the currently known age of Xiaohe Cemetery is about 3980 to 3540 yr BP, which was between early Xia (2070–1600 BC) to early Shang Dynasty (1600–1046 BC) in China, i.e. early Bronze Age. Many well-preserved mummies were found in this cemetery. Bergman described a lady with very strong European characters (e.g. brown hair, fine aquiline nose). DNA analysis from 30 mummies found here also demonstrated that the Xiaohe People were a West-East admixed population, which constitutes the earliest genetic evidence for an admixed population settled in the Tarim Basin [16].

## Materials and Methods

In 2003, we completed our archaeological research at the site of the Xiaohe Cemetery and collected four mud samples from each of the mud coffins (BM-1, BM-28, BM-75 and M-100) (Figure 2, 3 b) for laboratory study. Meanwhile, we also collected many leaves of *P* *. euphratica* in one of the tombs with poplar remains (Figure 3 c–f).

**Figure 4:**
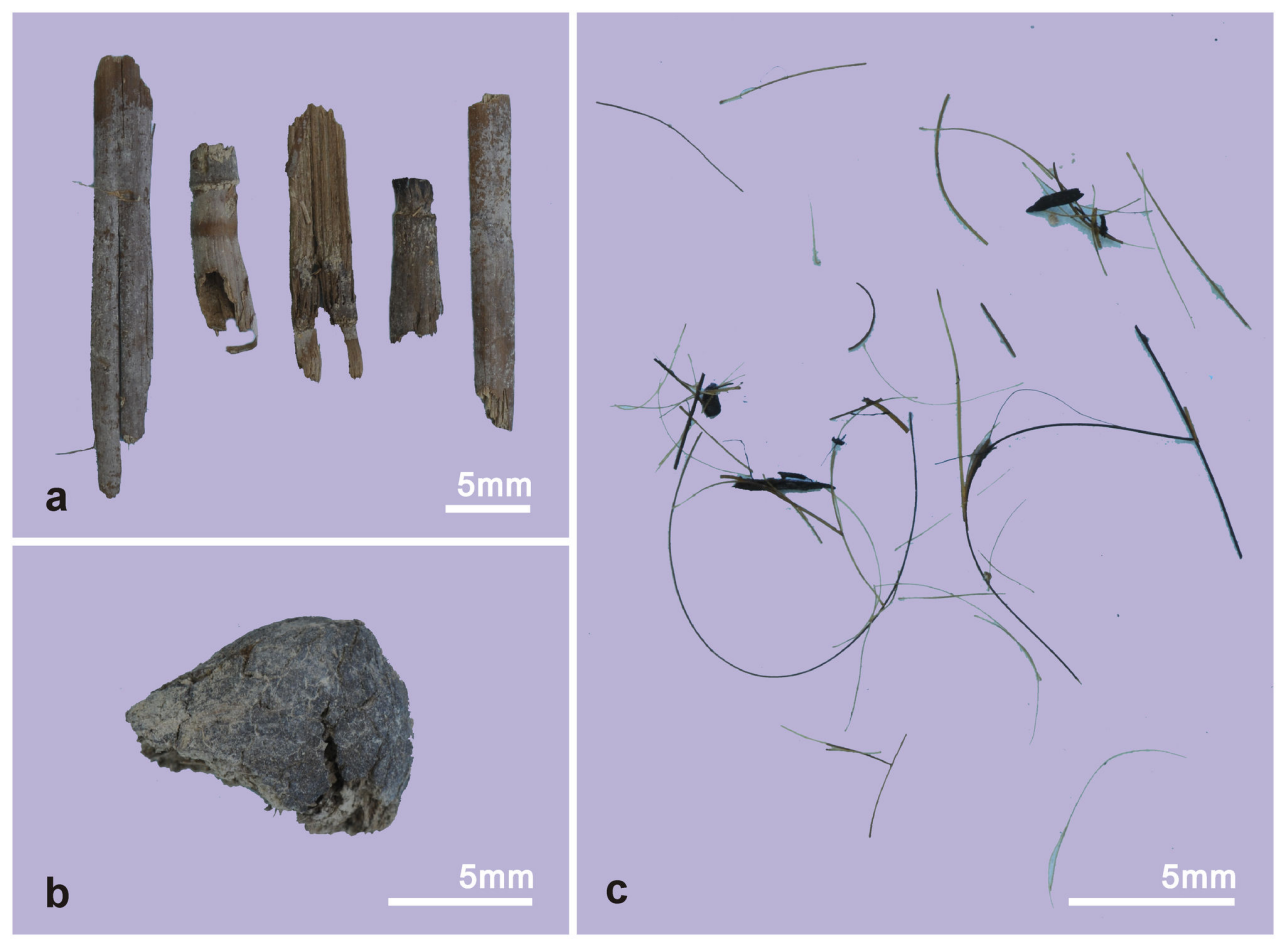
Other materials found from the samples. (a) straws; (b) piece of sheep manure; (c) livestock hairs.

In the laboratory, we first weighed 30 grams of each sample and put them into beakers with distilled water containing 1 milliliter of a suspension of *Lycopodium* spores (ca. 83,500 grains per milliliter). After immersion for 48 hours, the samples were sieved using a mesh with the pore size of 1 mm^2^. The residue was mainly composed of macrobotanical remains and livestock hairs (Figure 4 c). The screenings were prepared for a palynological study using the heavy liquid method [18]. We counted the contents of three slides from each specimen to obtain a representative sample of the palynomorphs (Table 1).

**Table 1:** Pollen counting data of the samples.

**Sample No.**	**Chenopodiaceae**	***Artermisia***	** *Ephedra* **	** *Corylus* **	***Alnus***	***Typha***	**Gramineae**	**Unknown**	**Lycopodium spores**
**BM1**	1	\	\	\	\	\	1	\	156
**BM28**	\	\	\	\	\	\	\	\	1189
**M75**	\	48	15	\	\	6	1	4	1057
**M100**	11	5	1	1	1	\	\	\	236

## Results and Discussion

### Palynological analysis

**Figure 5:**
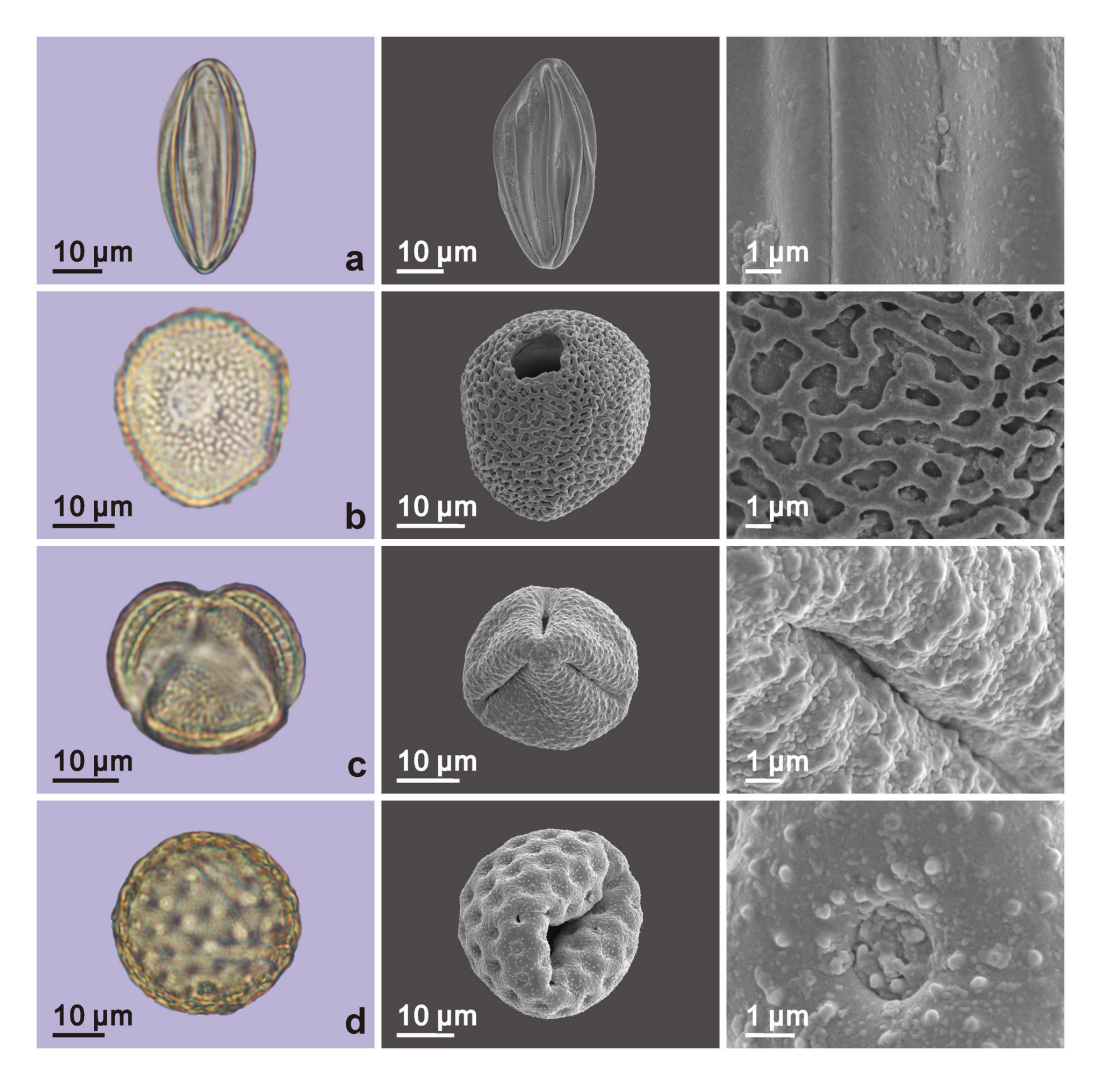
Palynomorphs found from the samples collected from the mud coffin. The first column shows pollen grains under light microscope; the middle column shows the previous grains under the scanning electronic microscope; and the last column shows the surface details under scanning electronic microscope. (a) *Ephedra* ; (b) *Typha*; (c) *Artemisia* ; (d) Chenopodiaceae.

**Figure 6:**
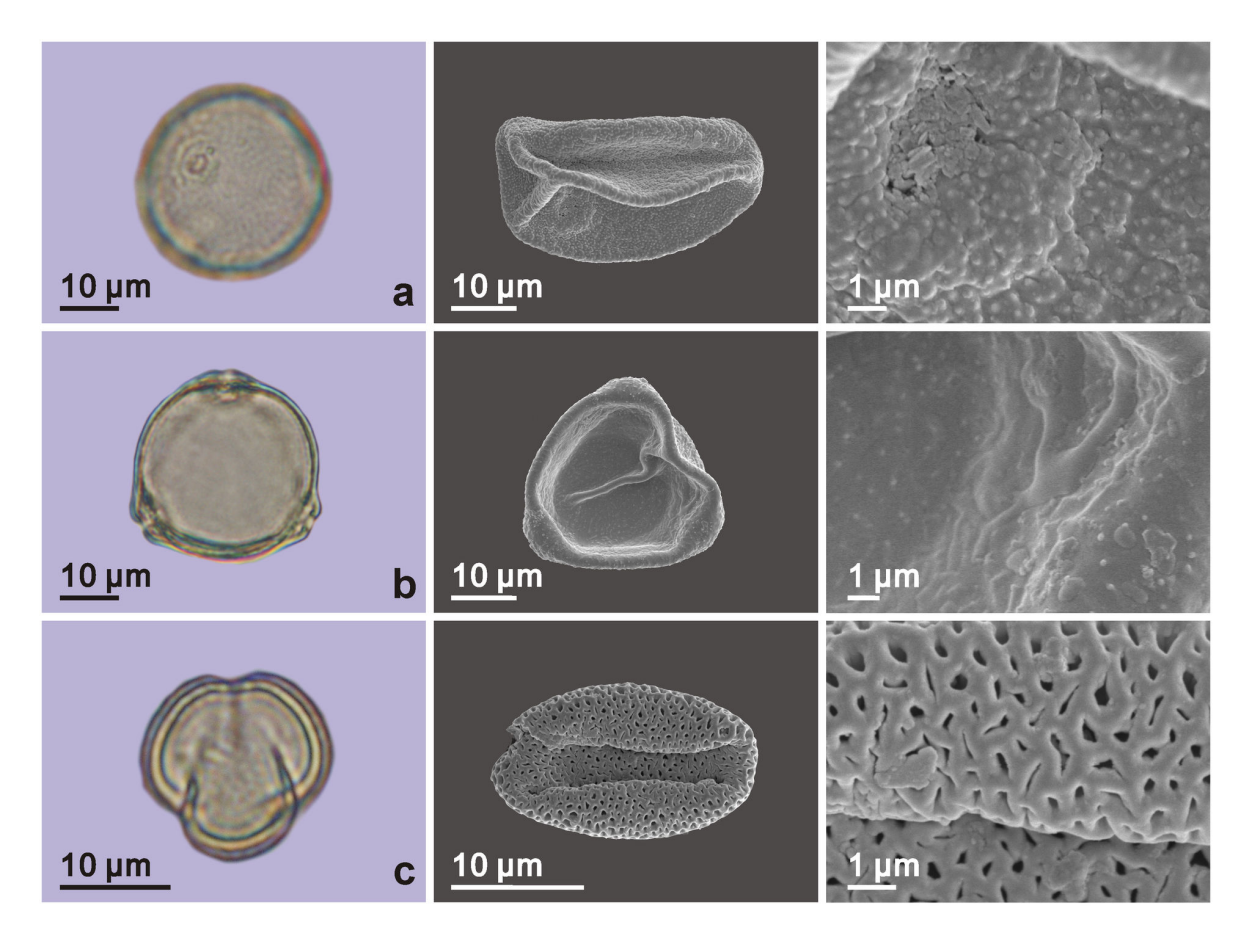
Palynomorphs found in the samples collected from the mud coffin. The first column shows pollen grains under light microscope; the middle column shows the previous grains under the scanning electronic microscope; and the last column shows the surface details under scanning electronic microscope. (a) Gramineae; (b) *Corylus* ; (c) *Tamarix* .

Totally, 96 pollen grains, belonging to nine types of palynomorphs, were found in the four palynological samples (Table 1) and all taxa were identified applying single-grain technique [19] (Figure 5, Figure 6; the only pollen grain of *Alnus* was lost during the preparation for the scanning electronic microscope). Most of the taxa are common in arid areas (e.g. Chenopodiaceae, *Artemisia* , *Ephedra* *,* *Tamarix* and Gramineae). Chenopodiaceae is well-adapted to dry and saline environments. *Artemisia* normally grows in arid or semi-arid habitats. *Ephedra* is a common shrub of dry, open sites and is predominantly a warm desert-steppe plant restricted to both meteorologically and physiologically dry areas [20]. *Tamarix* is one of the most common woody plants in Xinjiang [21]. Gramineae pollen grains are often used as an indicator of openness. However, these grass pollen are one of the most ubiquitous and readily recognized pollen types found in terrestrial sediments. Although these monoporate grains differ in size, surface texture and annular width, these features do not permit reliable recognition at the subfamily level [22]. Moreover, the pollen wall of Gramineae is thin, and is low in sporopollenin, hence has a poor preservational potential [23]. These factors suggest that the pollen cannot be transported far and are easily damaged when buried in soil. However, based on the number of reeds ( *Phragmites* sp.) found in the graves, it seems reasonable to believe that most of the Gramineae pollen in our samples belong to *Phragmites* . *Corylus* and *Alnus*, as wind-pollinated taxa very readily overproduce airborne pollen grains, and are the normal elements found in lake sediments and soil. The grains of these two taxa may come from the forests on the mountains surrounding the study areas.

Compared with the taxa mentioned above, the presence of *Typha* pollen in the assemblage is more interesting. As an aquatic, *Typha* can normally live in a variety of wetland habitats, and their pollen grains can be frequently found in peat and lignite beds [24]. Hence the occurrence of *Typha* pollen illustrates that there was surface water close to the Xiaohe People’s residence. The mud which covered the coffins may have been obtained from the habitat of the *Typha*.

### Plant remains

At a traditional funeral in China, people always put something precious into the coffin and/or grave, in the hope that the dead person can live better with these objects in another world. Those relics found in tombs provide us with an opportunity to learn about the culture of the ancient people and the environment in which they lived.

Several kinds of plant remains were found during the first excavation of the cemetery by Bergman, i.e. the poles made of *Populus* *euphratica* *,* *Ephedra* twigs, *Tamarix* twigs, grains of bread wheat (*Triticum aestivum*) [17], Jiji grass ( *Achnatherum* *splendens* ), reed ( *Phragmites* sp.) and grains of broomcorn millet ( *Panicum* *miliaceum* ) [13,14,17]. DNA analysis of the wheat grains confirms that the grains found here are similar to hexaploid bread wheat [17]. As broomcorn millet grains always show up together with wheat grains in the graves [14,17], we can presume that broomcorn millet was also cultivated by then. *Populus* *euphratica* is the characteristic element of the common desert riparian forest in Northwest China [25]. As a common shrub with medicinal function, *Ephedra* was considered as a magic plant by the Lop people. Also, it is very common to find *Ephedra* branches in most of the graves of the ancient Lop people in the Lop Nur area, such as LF, LS and LD graveyards [26], Cemetery 36 [26], Gumugou cemetery [27] and graveyards around Loulan ancient city [28]. Some Chinese archaeologists suggest that this phenomenon is a kind of plant worship and call it ephedra worship [27,28]. The medical use of *Ephedra* has been known for several thousand years in China. As a central nervous excitant, ephedra was also used in ceremonies to produce feelings of exhilaration by various religious groups including Hindus [29]. As an ingredient of Haoma or Soma, ephedra has been used for millennia in both Iran and India [30] as a beverage to achieve longevity and immortality [31]. The intention of putting tamarisk twigs in the burials has never been studied yet. The grains of wheat (*Triticum aestivum*) were normally found together with the ephedra twigs (or fragments) in graves [28]. Moreover, some dried porridge of millet was also preserved in some graves [13]. These plant remains indicate that wheat and millet were also very precious for the ancient people.

### Other plant and animal matter

The straws (Figure 4 a) found in the mud samples were used to reinforce the mud for construction purposes. This technique is still widely used in the countryside of China. These straws may have originated from wheat and/or millet. The appearance of the livestock hairs (Figure 4 b) and the sheep manure (Figure 4 c) in the samples illustrates that the earth used to make the covering layer of the coffins must have been obtained from a place frequented by the animals. The occurrence of many/numerous bones and furs in the Xiaohe Cemetery [13,14], suggests that some of the Xiaohe People were living as herdsmen.

### Environmental analysis

Based on the plant analyses presented above, the presence of both xerophytic and hydrophytic plants (e.g. *Typha*) demonstrate that there was enough water in the Small River at that time though it lies in the expansive Lop Desert.

In the surrounding desert, there were many *Ephedra* , *Tamarix* , *Artemisia* and some members of the Chenopodiaceae plants. The people apparently collected the ephedra and *Tamarix* for medicinal or religious use from the neighboring arid terrains. However when the people lived there the site was a well-watered wetland along the Small River. There the rich alluvial soils of the flooded areas served to support the growth of their crops, and provide areas where livestock could be sustained/raised. Moreover, this location was the habitat of the common *Populus* *euphratica* which served in the construction of their houses and coffins. So although the regional natural environment was very dry, the hydrological living conditions were good enough along the small river for the Xiaohe People to survive.

## Conclusions

Much research has shown that the climate in the Lop Nur region has been very dry since the Early Holocene [32–34]. However, the so-called dry climate is actually a kind of meteorological myth. Fed by melt-water from the Tianshan Mountain, the runoffs of the rivers into the Tarim Basin are actually quite considerable, especially in summer time. Many oases depend on such seasonal rivers. During 3600-3000 a BP, the lake of Lop Nur was very large and there were many deltas around it. Fishing and hunting were very common at that time [35]. According to historical documents, the water area of Lop Nur was still very large during the Jin Dynasty (AD 226-420) [36].

Based on this work, the living environment of the Xiaohe People was a very well developed oasis of deltas, which was surrounded by extensive desert. The main taxa of the vegetation in the oasis were *Populus* *euphratica* , *Phragmites* *, Typha* and maybe other weedy Gramineae. However, outside the oasis, drought-resistant taxa dominated the vegetation, e.g. *Ephedra* , *Tamarix* , *Artemisia* and members of the Chenopodiaceae.

The Xiaohe People mainly lived on animal husbandry. However, they also attempted to cultivate cereals such as bread wheat and broomcorn millet. Most of the coffins in the cemetery are canoe-shaped, which may suggest that the Xiaohe People spent some of their lives on water. Bergman [13] inferred that because there are no known settlements near the cemetery, the people probably lived somewhere else along the river and reached the cemetery by boats.
